# Association between different anti-Tat antibody isotypes and HIV disease progression: data from an African cohort

**DOI:** 10.1186/s12879-016-1647-3

**Published:** 2016-07-22

**Authors:** Francesco Nicoli, Mkunde Chachage, Petra Clowes, Asli Bauer, Dickens Kowour, Barbara Ensoli, Aurelio Cafaro, Leonard Maboko, Michael Hoelscher, Riccardo Gavioli, Elmar Saathoff, Christof Geldmacher

**Affiliations:** Center for International Health, Ludwig-Maximilians-Universität München, Leopoldstraße 7, 80802 Munich, Germany; Department of Life Sciences and Biotechnology, University of Ferrara, Ferrara, Italy; National Institute for Medical Research (NIMR)-Mbeya Medical Research Centre, Mbeya, Tanzania; Division of Infectious Diseases and Tropical Medicine, Medical Center of the University of Munich (LMU), Munich, Germany; National AIDS Center, Istituto Superiore di Sanità, Rome, Italy; German Center for Infection Research (DZIF), partner site Munich, Munich, Germany; Current address: CIMI INSERM U1135, 91 bd del’Hopital, 75013 Paris, France

**Keywords:** Tat, Antibodies, Diseases progression, Clade B HIV, Clade C HIV, Immune activation

## Abstract

**Background:**

The presence of IgG and IgM against Tat, an HIV protein important for viral replication and immune dysfunction, is associated with slow disease progression in clade B HIV-infected individuals. However, although Tat activities strictly depend on the viral clade, our knowledge about the importance of anti-Tat antibodies in non-clade B HIV infection is poor. The objective of this study was to investigate the association of different anti-Tat antibody isotypes with disease progression in non-clade B HIV-infected subjects and to study the relationship between anti-Tat humoral responses and immunological abnormalities.

**Methods:**

Anti-clade B and -clade C Tat IgG, IgM and IgA titers were assessed in serum samples from 96 cART-naïve subjects with chronic HIV infection from Mbeya, Tanzania, and associated with CD4^+^ T cell count, plasma viremia and CD4^+^ and CD8^+^ T cell phenotypes.

**Results:**

Anti-Tat IgM were preferentially detected in chronic HIV-infected subjects with low T cell activation (*p-v*alue = 0.03) and correlated with higher CD4^+^ T cell counts and lower viral loads irrespective of the duration of infection (*p-*value = 0.019 and *p-*value = 0.037 respectively). Conversely, anti-Tat IgA were preferentially detected in individuals with low CD4^+^ T cell counts and high viral load (*p-*value = 0.02 and *p-*value < 0.001 respectively). The simultaneous presence of anti-Tat IgG and IgM protected from fast CD4^+^ T cell decline (*p-*value < 0.01) and accumulation of CD38^+^HLADR^+^CD8^+^ T cells (*p-* value = 0.029).

**Conclusions:**

Anti-Tat IgG alone are not protective in non-clade B infected subjects, unless concomitant with IgM, suggesting a protective role of persistent anti-Tat IgM irrespective of the infecting clade.

**Electronic supplementary material:**

The online version of this article (doi:10.1186/s12879-016-1647-3) contains supplementary material, which is available to authorized users.

## Background

The HIV Tat protein is fundamental for HIV infection, replication and dissemination. Tat is a transcriptional transactivator of the HIV genome [1] which favours the generation of new activated CD4^+^ T cell targets for infection [2–4] and interacts with Env enhancing viral infectivity [5, 6]. Tat activity also induces the release of pro-inflammatory cytokines and up-regulation of transcription factors involved in T cell activation contributing to hyperactivation and dysfunction of T cells [2, 3, 7–10]. Moreover, Tat interacts with various co-infecting opportunistic pathogens [11] and is directly implicated in the pathogenesis of AIDS-related Kaposi’s sarcoma [12], several vasculopathic conditions [13] and HIV-associated dementia [14]. Interestingly, the effects of Tat differ depending on the HIV clade [14–16].

Anti-Tat immunity might counteract the Tat-mediated immune dysregulation and hence play a role in controlling HIV infection and co-morbidity. Previous studies showed that anti-Tat IgM and IgG, although present in a small proportion of HIV-infected individuals, are more frequently found in the asymptomatic stage of infection [17, 18] and in non-progressors [19] and are associated with maintenance of CD4^+^ T cell counts [20–22] and low viral load [23, 24]. However, most of these studies were conducted in clade B HIV-infected cohorts and with clade B Tat, whereas the effect of naturally occurring anti-Tat antibodies in non-clade B HIV infections has been poorly investigated and the relationship between anti-Tat humoral responses and the development of immunological abnormalities has not been reported.

In this study, a comprehensive analysis of different anti-Tat antibody isotype levels was conducted to investigate the association of anti-Tat IgG, IgA and IgM with CD4^+^ T cell count, viral load and immunological abnormalities in chronically non-clade B HIV-infected individuals.

## Methods

### Study design

Human serum samples from 96 cART-naïve chronically HIV-infected adults (minimum duration of infection of >1 year) from the “Worm_HIV_Interaction_Study” (WHIS) cohort, Mbeya Medical Research Center, Mbeya, Tanzania, were included in the analyses. Characteristics of study subjects are shown in Table 1. The WHIS cohort study is described in detail elsewhere [25].

**Table 1:** Characteristics of the HIV positive individuals included in the study (*n* = 96^a^)

Age^b^		36.1 (28.8–42.3)
Female, *n* (%)		58 (60.4 %)
CD4^+^ T cell counts (cells/μl)^b^		398 (267–606)
Log_10_ pVL (copies/ml)^b^		4.7 (4.0–5.3)
Duration of infection (n)^c^	>1 year, < 3 years	20
	>3 years	69

^a^Not all 96 subjects had data for each of the examined parameters (CD4, *n* = 95; pVL, *n* = 89)

^b^Values shown are median and (Interquartile Range)

^c^Duration of infection could be confidentially estimated for 89 subjects out of 96

Absolute CD4^+^ T cell counts were determined from anti-coagulated whole blood using a BD FACS Multitest IMK kit (BD) according to manufacturer instructions. HIV-1 plasma RNA concentrations were measured in plasma samples of HIV positive subjects using either the Cobas Amplicor HIV-1 Monitor Test version 1.5 or Cobas Taqman 48 analyzer (Roche Diagnostics).

### Enzyme-linked immunosorbent assay (ELISA)

Human anti-Tat IgG, IgM and IgA were measured in sera by ELISA [17, 22, 26–29] using sera collected during the baseline visit. Ninety-six-well immunoplates (Nunc Max Sorp) coated with 100 ng/well of clade B or clade C Tat (Diatheva, Additional file 1) resuspended in 0.05 M sodium carbonate buffer (pH 9.6–9.8), for 16 h at 4 °C. Plates were washed five times with PBS (pH 7.0) containing 0.05 % Tween-20 (Sigma) and then blocked with PBS containing 0.05 % Tween 20 and 1 % BSA for 90 min at 37 °C (IgG) or 60 min at room temperature (IgA) or with PBS containing 5 % milk for 60 min at 37 °C (IgM). Plates were washed five times and 100 μl/well of appropriate dilutions of each serum diluted in PBS containing 0.05 % Tween 20 and 1 % BSA (“blocking buffer” IgG and IgA) or PBS containing 5 % milk (“blocking buffer” IgM) were dispensed in duplicate wells and then incubated for 90 min at 37 °C. Plates were washed again before the addition of 100 μl/well of HRP-conjugated anti-human IgG (Sigma), diluted 1:1000, or HRP-conjugated anti-human IgA (Sigma) diluted 1:3000, or HRP-conjugated anti-human IgM (Sigma), diluted 1:1000, in the appropriate blocking buffer and incubated at 37 °C for 90 min (IgG) or for 60 min (IgA and IgM). In each plate, two wells were incubated with blocking buffer plus secondary antibodies (blank). After incubation, plates were washed five times and incubated with blocking buffer for 15 min at 37 °C (step performed only for IgG and IgM). Plates were washed five times and then incubated with ABTS (Roche) for 50 min after which time absorbance values were measured at 405 nm with an automatic plate reader (SUNRISE TECAN). The cut-off value was estimated as the mean absorbance of 3 negative control sera plus 0.05. Negative control sera were randomly selected from HIV-negative subjects enrolled in the WHIS cohort. Blank and cut-off values were subtracted from the absorbance value of each sample to obtain net absorbance values. To determine the presence of anti-Tat antibodies, all sera were first screened at a dilution of 1:100 for IgG and 1:25 for IgA and IgM. Then positive sera (net absorbance > 0) were titrated using serial 2-fold dilutions. Serum samples with anti-Tat IgG, IgA and IgM were considered positive if antibody titers were ≥ 100, 25 and 25 respectively. Titers were calculated by intercept function using the Excel program (Microsoft).

ELISA assays were performed in a blinded way with respect to immunological parameters and progression markers.

### Flow cytometry

The proportion of T cells expressing activation (HLA-DR and CD38) and maturation (CD27 and CD45R0) markers was determined in fresh, anti-coagulated whole blood as previously described [25]. Briefly, fresh blood samples were incubated for 30 min using the following fluorochrome-labelled monoclonal antibodies (mABs): CD3-Pacific Blue (BD), CD4 Per-CP Cy5.5 (eBioscience), CD8 V500 or CD8 Amcyan, CD27 APC-H7, CD45RO APC, HLA-DR FITC and CD38 PE (all from BD). Acquisition was performed on a FACS CANTO II (BD). Compensation was conducted with antibody capture beads (BD) stained separately with the individual antibodies used in the test samples. Flow cytometry data was analysed using FlowJo (version 9.5.3; Tree Star Inc.).

### Activation burden

The “activation burden” reported in this study refers to a composite measure of T cell abnormalities defined by the presence of 0, 1-2, 3 or more abnormalities of the T cell phenotype. This approach was similar to that used to define the “inflammatory burden” in HIV-infected individuals [30].

The correlation of CD4^+^ T cell counts and plasma viral load (pVL) with the percentages of CD8^+^ and CD4^+^ T cell subpopulations (single or double expression of HLA-DR/CD38 or CD45RO/CD27) and the values of CD4:CD8 ratio was assessed, and only those phenotypes that significantly correlated (*P*-value Spearman’s correlation ≤0.05, not shown) with disease progression (defined as the simultaneous decrease of CD4^+^ T cells number and increase of pVL) were chosen as parameters to define the “activation burden”. These parameters were the percentages of CD4^+^HLA-DR^+^CD38^+^ and CD8^+^HLA-DR^+^CD38^+^ T cells (inverse correlation with CD4^+^ T cell counts and direct correlation with pVL), the percentages of CD4^+^CD45RO^−^CD27^+^ and CD8^+^CD45RO^−^CD27^+^ T cells and the CD4:CD8 ratio (direct correlation with CD4^+^ T cell counts and inverse correlation with pVL).

To assign scores, each parameter was divided into quartiles named from A to D, where A indicated the most abnormal values, mentioned above that were associated with disease progression. Accordingly, for each parameter, quartile “A” included individuals with the highest proportion of CD4^+^HLA-DR^+^CD38^+^ and CD8^+^HLA-DR^+^CD38^+^ T cells, the lowest proportion of CD4^+^CD45RO^−^CD27^+^ and CD8^+^CD45RO^−^CD27^+^ T cells, as well as the lowest CD4:CD8 ratio. Conversely, quartile “D” included subjects with the opposite values for each parameter, all positively correlated with CD4^+^ T cell counts and negatively with pVL. To determine the activation burden, the number of “A” was calculated for every donor: for example, an activation burden score of 0 corresponds to having none of the five phenotype parameters at abnormal levels while the score of 3 was defined as having three or more parameters at abnormal levels.

### Statistical analysis

Data analyses were performed using Prism version 5 (GraphPad Inc.), Microsoft Excel (Microsoft) and Stata version 13 (StataCorp, TX). Groups were compared using the Mann-Whitney *U*-test, the Wilcoxon signed rank test or Fisher’s exact test as appropriate. For association analyses, the Spearman rank correlation was determined. *P-*values ≤ 0.05 were regarded as significant. Associations of different anti-Tat antibody isotypes with CD4^+^ T cell count and viral load were calculated using uni- and multi-variate Poisson regression with robust variance estimates. Figure and table legends describe which test was used in which case. Heatmaps were created and hierarchical clustering performed with Qlucore Omics Explorer 3.2.

## Results

### Prevalence of anti-Tat humoral responses in chronically HIV-infected individuals

**Fig. 1:**
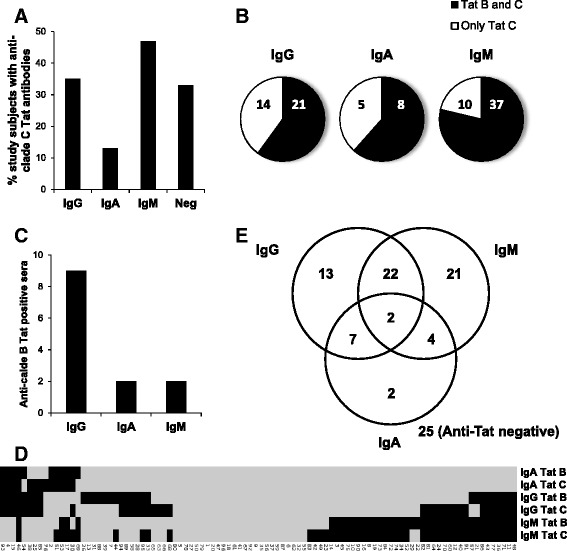
Prevalence and cross-clade reactivity of anti-Tat antibodies. CART-naïve, chronically HIV-infected individuals (*n* = 96) were tested for the presence of anti-Tat antibodies by ELISA. **a** Percentage of subjects with anti-clade C Tat IgG, IgA or IgM or anti-clade C Tat negative. **b** Number of anti-clade C Tat positive individuals able to recognize clade B Tat. **c** Number of subjects able to recognize clade B but not clade C Tat. **d** Heatmap showing, for single donors, positivity (black) or negativity (grey) toward clade C or clade B Tat for each isotype after unsupervised clustering. **e** Venn diagram analyses of the number of subjects recognizing clade B and/or C Tat stratified by the antibody isotype displayed

Previous studies have shown that Mbeya Region is affected by a multi-clade epidemics characterized by a high proportion of clade C and AC recombinant forms of HIV as well as a small fraction of clade D-containing recombinant forms, while clade B is absent [31–33]. Thus, serum samples were first tested for the presence of antibodies recognizing clade C Tat. Anti-Tat IgG were detected in 36 % of HIV-1 infected subjects, anti-Tat IgA in 14 % and anti-Tat IgM in 49 %. Thirty four % of individuals did not have serum antibodies recognizing clade C Tat (Fig. 1a). We then assessed the capacity of the anti-Tat antibodies in this non-clade B cohort to recognize clade B Tat. As shown in Fig. 1b, 21 of the 35 (60 %) subjects with detectable anti-clade C Tat IgG also recognized clade B Tat. Similar proportions were found for anti-Tat IgA (8 of 13, 62 %), while 37 of the 47 (79 %) subjects with anti-Tat C IgM recognized clade B Tat, indicating that IgM displayed the highest cross-reactivity. Notably, some HIV-1 infected individuals recognized clade B but not clade C Tat (Fig. 1c,d and Additional file 2). Overall, 71 of 96 subjects (74 %) had detectable antibodies against Tat (clade C and/or B): 44 had IgG, 15 IgA and 49 IgM (Fig. 1e and Additional file 2). Twenty-five out of the 49 anti-Tat IgM responders did not have anti-Tat IgG, suggesting a lack of isotype switch despite chronic infection. In contrast, anti-Tat IgA were mostly found associated with other isotypes, as only 2 of the 15 anti-Tat IgA responders exclusively mounted IgA responses (Fig. 1d). These two subjects might have lost IgG and/or IgM over time, although the presence of IgA in the absence of other isotypes has been reported in HIV infection [34, 35].

Together, these analyses demonstrate that a large proportion of the 96 chronically HIV-infected adults had a serum anti-Tat antibody response dominated by IgG or IgM.

### Anti-Tat IgM and IgA are differently associated with CD4+ T cell count and plasma viremia

To determine the association between anti-Tat antibody isotypes and CD4^+^ T cell count, we evaluated the frequencies of subjects with the different anti-Tat isotypes after stratification according to the immunological HIV staging [36]. For each anti-Tat isotype, responders were considered as those individuals that were positive to at least one of the two clades (C and/or B). As shown in Table 2, anti-Tat IgG were present at a similar frequency in the three groups (>500, 500-200, <200 CD4^+^ T cells/μl), while anti-Tat IgA were detected at higher frequency in subjects with low CD4^+^ T cell counts. A significantly higher prevalence of anti-Tat IgM was detected in individuals with CD4^+^ T cell counts greater than 500 cells/μl (*p-*value = 0.008). Anti-Tat antibody-negative subjects were more likely to have low CD4^+^ T cell counts, although this difference was not significant (*p-*value = 0.27). Since these data suggest that anti-Tat IgM responses are associated with a better disease prognosis, and usually IgM responses tend to disappear over time [37–39], we sought to investigate whether the presence of this isotype and its association with high CD4^+^ T cell counts was dependent on the duration of HIV infection. Thus, subjects were stratified according to the duration of infection: less (*n* = 20) or more (*n* = 69) than 3 years. For 7 subjects the duration of infection could not be established and were therefore excluded from this analysis.

**Table 2:** Frequency of anti-Tat antibodies and disease stage

		IgG	IgA	IgM	Negative
CD4^+^ T cells (cells/μl)	<200	7/17 (41 %)	4/17 (24 %)	5/17 (29 %)	7/17 (41 %)
	200–500	23/48 (48 %)	10/48 (21 %)	22/48 (46 %)	12/48 (25 %)
	>500	14/30 (47 %)	1/30 (3 %)	22/30 (73 %)	6/30 (20 %)
	*p*-value^a^	0.92	0.05	0.008	0.27

^a^Frequency of the different anti-Tat antibody isotypes was calculated in every stratum and compared using Freeman-Halton extension of the Fisher’s exact probability test for a two-rows by three-columns contingency table

As shown in Table 3, the proportion of anti-Tat IgG responders was comparable irrespective of the infection duration, while IgA were rarely detected in individuals infected for less than 3 years. Interestingly, IgM were detected in 75 % of individuals infected for less than 3 years and still maintained in 45 % of subjects infected for more than 3 years. Multivariate analysis revealed that the duration of infection did not affect the association between the presence of anti-Tat IgM, high CD4^+^ T cell counts and low viral load and between the presence of anti-Tat IgA, CD4^+^ T cell loss and high viral load (Table 4).

**Table 3:** Frequency of anti-Tat antibodies according to the duration of infection

		IgG		IgA		IgM	
Time of infection		< 3 years	> 3 years	< 3 years	> 3 years	< 3 years	> 3 years
Anti-Tat antibodies status	+	8/20 (40 %)	32/69 (46 %)	1/20 (5 %)	14/69 (20 %)	15/20 (75 %)	31/69 (45 %)
	−	12/20 (60 %)	37/69 (54 %)	19/20 (95 %)	55/69 (80 %)	5/20 (25 %)	38/69 (55 %)
*p*-value^a^		0.80		0.17		0.02	

^a^Frequency of the different anti-Tat antibody isotype was calculated in every stratum and compared using Fisher’s exact probability test

**Table 4:** Association of different anti-Tat antibody isotypes with CD4^+^ T cell count and viral load

Association^a^ of duration of HIV infection and anti-Tat antibody isotype with CD4^+^ T cell counts (*n* = 88)									
					Univariate			Multivariate	
Covariate	Stratum	*n*	Mean CD4^+^ T cells count (cells/μL)	IRR	95 % conf.int.	*p*-value	IRR	95 % conf.int.	*p*-value
HIV infection (years)	>3	69	403	1	–	–	1	–	–
	≤3	19	501	1.24	(0.98 to 1.58)	0.079	1.10	(0.86 to 1.41)	0.443
IgG	neg.	48	431	1	–	–	1	–	–
	pos.	40	415	0.96	(0.76 to 1.22)	0.764	0.97	(0.77 to 1.21)	0.761
IgM	neg.	43	353	1	–	–	1	–	–
	pos.	45	491	1.39	(1.10 to 1.76)	0.007	1.34	(1.05 to 1.71)	0.019
IgA	neg.	73	448	1	–	–	1	–	–
	pos.	15	306	0.68	(0.51 to 0.91)	0.010	0.73	(0.55 to 0.95)	0.020
Association^a^ of duration of HIV infection and anti-Tat antibody isotype with Log_10_ VL (*n* = 83)									
					Univariate			Multivariate	
Covariate	Stratum	*n*	Mean viral load (Log_10_ copies/ml)	IRR	95 % conf.int.	*p*- value	IRR	95 % conf.int.	*p*-value
HIV infection (years)	>3	65	4.68	1	–	–	1	–	–
	≤3	18	4.26	0.91	(0.79 to 1.05)	0.203	0.96	(0.83 to 1.10)	0.553
IgG	neg.	45	4.47	1	–	–	1	–	–
	pos.	38	4.74	1.06	(0.96 to 1.16)	0.230	1.05	(0.96 to 1.15)	0.284
IgM	neg.	40	4.84	1	–	–	1	–	–
	pos.	43	4.36	0.90	(0.82 to 0.99)	0.027	0.92	(0.85 to 0.99)	0.037
IgA	neg.	68	4.46	1	–	–	1	–	–
	pos.	15	5.20	1.17	(1.08 to 1.26)	<0.001	1.13	(1.06 to 1.21)	<0.001

*IRR* incidence rate ratio

^a^Poisson regression with robust variance estimates was used to examine the association of Tat antibody isotypes with CD4^+^ T cell count and viral load. The table shows that the duration of HIV infection and anti-Tat IgG positivity had no significant influence on both parameters, whereas the presence of anti-Tat IgM and IgA were significantly associated with CD4^+^ T cell count and viral load. Anti-Tat IgM positivity was associated with higher CD4^+^ T cell counts and lower viral loads, whereas anti-Tat IgA positivity showed the opposite pattern. The fact that multivariate IRRs and p-values for both parameters are fairly similar to the univariate estimates suggest that these associations are independent of each other

To better understand the association of the different anti-Tat isotypes with CD4^+^ T cell numbers, subjects were stratified into 5 different groups: those displaying only IgM, only IgG, both IgG and IgM, IgA (irrespective of the presence of other isotypes, due to the low number of samples), and those who were anti-Tat antibody negative (see Table 5).

**Table 5:** Stratification of subjects according to anti-Tat antibody isotypes

		Antibody response (n)						Age: median (range)	Females
Isotype	(*n*)	IgG	IgG	IgA	IgA	IgM	IgM		
		Tat B	Tat C	Tat B	Tat C	Tat B	Tat C		*n* (%)
IgG	(13)	10	6	0	0	0	0	37 (26–45)	9 (69 %)
IgA	(15)	8	9	10	13	4	6	40 (25–55)	6 (40 %)
IgM	(21)	0	0	0	0	17	19	34 (20–54)	16 (80 %)
IgM + IgG	(22)	12	19	0	0	18	22	36 (20–61)	13 (59 %)
Neg	(25)	0	0	0	0	0	0	35 (19–53)	14 (56 %)

**Fig. 2:**
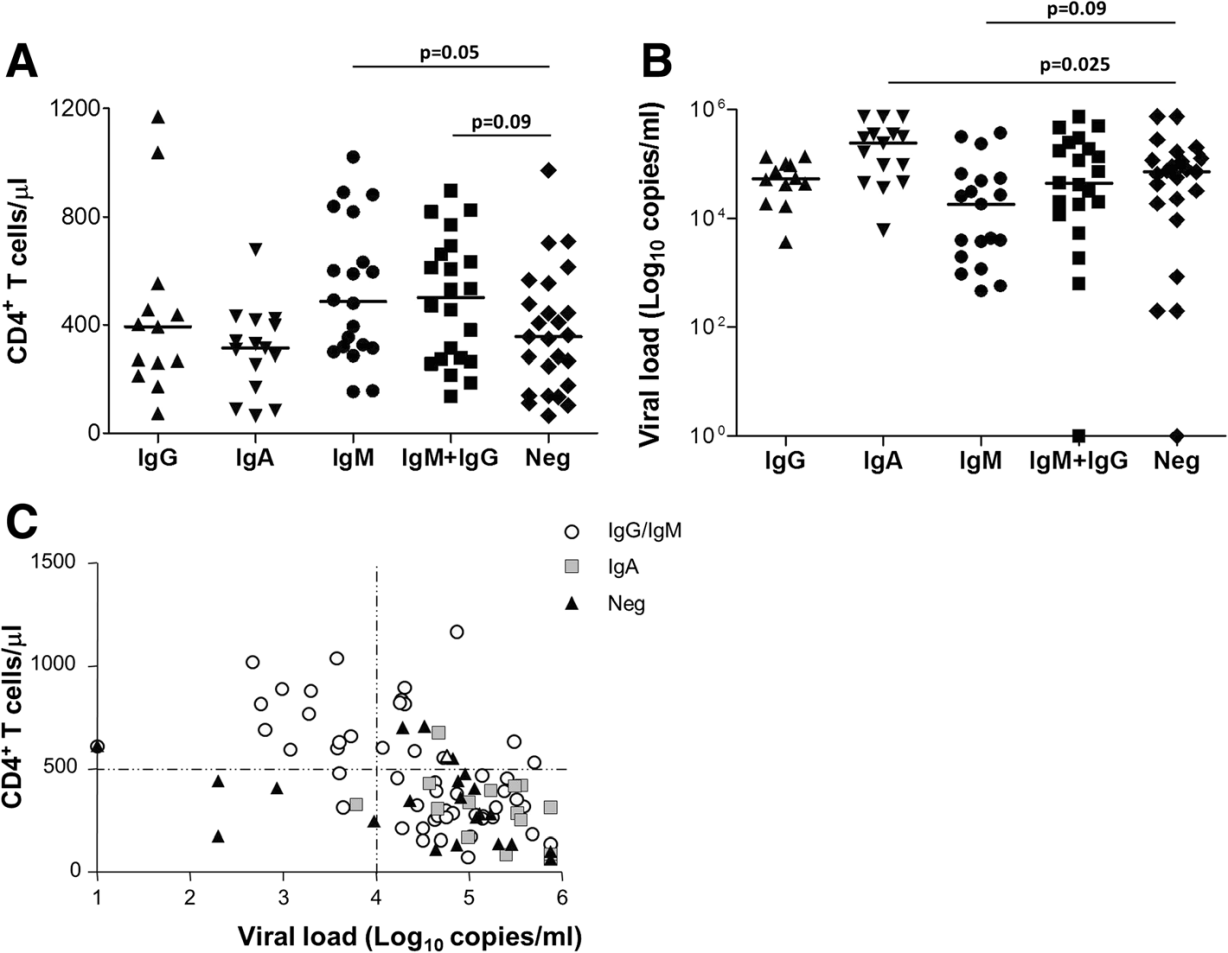
Association of anti-Tat antibody isotypes with CD4^+^ T cell count and viral load. Subjects were stratified according to the type and number of the anti-Tat antibody isotypes detected. Subgroups were compared for (**a**) CD4^+^ T cell count or (**b**) Log10 plasma viral load. Lines represent the median value. Statistical comparisons were made using the Mann-Whitney test. **c** Subjects were plotted according to both CD4^+^ T cell count and Log10 plasma viral load. Empty circles represent subjects with anti-Tat IgG and/or IgM but not IgA, grey squares represent subjects with anti-Tat IgA, black triangles represent anti-Tat antibody negative subjects

As shown in Fig. 2a, subjects with only anti-Tat IgM displayed significantly higher CD4^+^ T cell counts (median 486 cells/μl) compared to anti-Tat antibody negative individuals (median 356 cells/μl, *p-*value = 0.05). The simultaneous presence of anti-Tat IgM and IgG was also associated with a trend towards higher CD4^+^ T cell counts (median 501 cells/μl).

We next explored the association of the different anti-Tat antibody isotypes with plasma viral load (pVL). IgA responders had significantly higher levels of plasma viremia (median Log: 5.4 copies/ml) compared to anti-Tat antibody negative individuals (median Log: 4.9 copies/ml, *p-*value = 0.025, Fig. 2b), while subjects possessing only anti-Tat IgM had the lowest median viral load (median Log: 4.3 copies/ml). The majority of individuals with high CD4^+^ T cell counts (>500 cells/μl) and low pVL (<10^4^ copies/ml) displayed anti-Tat IgG and/or IgM, while anti-Tat antibody negative subjects or anti-Tat IgA responders had lower levels of CD4^+^ T cells and higher pVL (Fig. 2c).

These results show that anti-Tat IgM antibodies, alone or in combination with anti-Tat IgG, although declining with time, are still detectable after 3 years of HIV infection in many individuals and are associated with slow-progression, while serum anti-Tat IgA appears later and are associated with disease progression.

### Association between anti-Tat antibodies and T cell activation

HIV disease progression is characterized by several T cell abnormalities such as immune activation and loss of naïve T cells. Thus, an “activation burden” was defined based on those parameters that correlated with disease progression: the percentages of HLA-DR^+^CD38^+^ CD4^+^and CD8^+^ T cells, the frequency of CD45RO^−^CD27^+^ (naïve-like) CD8^+^ and CD4^+^ T cells and the CD4:CD8 ratio (see Additional file 3 for gating strategy).

**Fig. 3:**
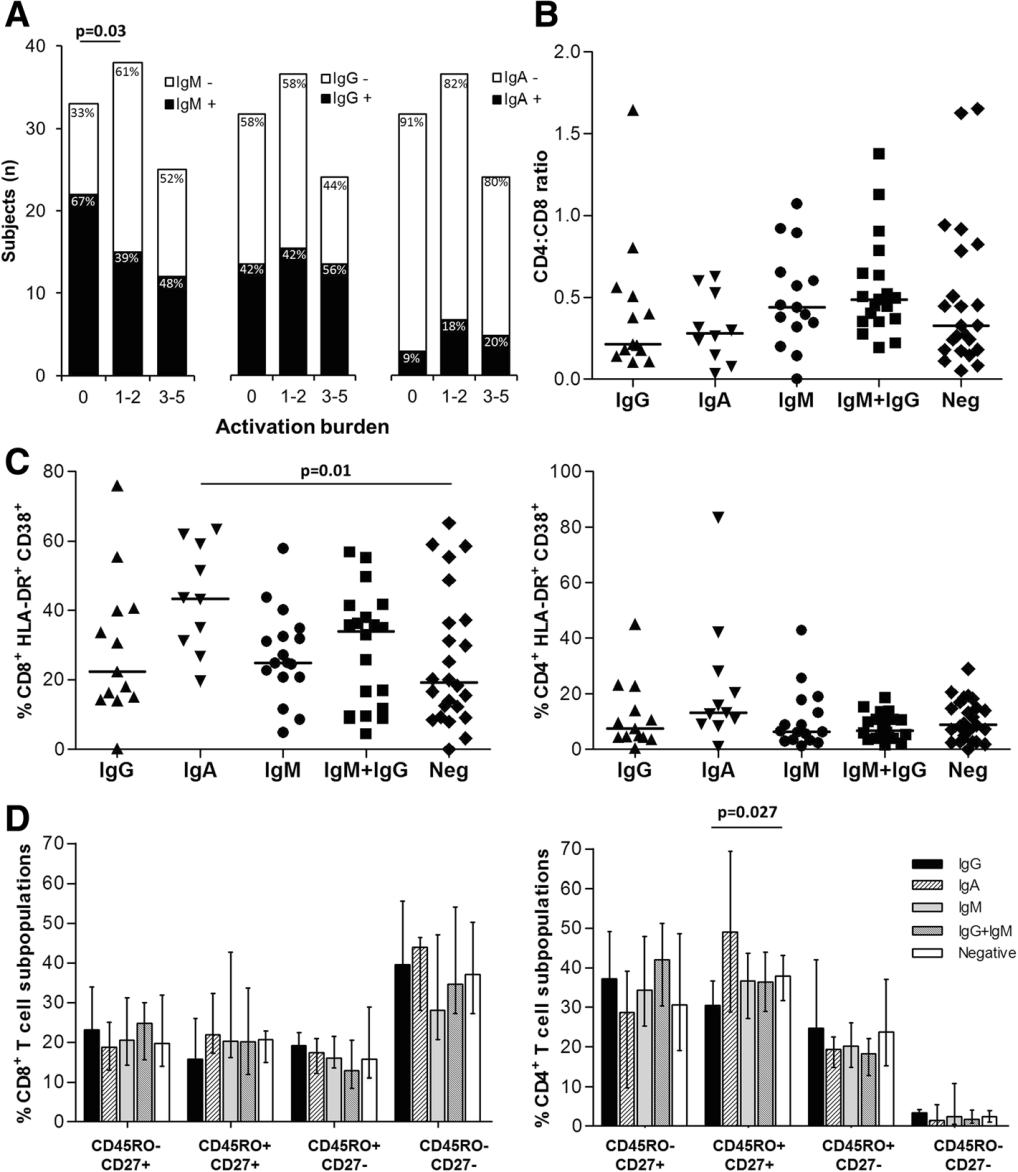
Association of anti-Tat antibody isotypes with immunological abnormalities. **a** Subjects were stratified according to the activation burden. Subjects in panels (**b**-**d**) were stratified according to the type and number of anti-Tat antibody isotypes. **b** Subgroups were compared for CD4:CD8 ratio. **c** Subgroups were compared for percentages of CD38^+^ HLA-DR^+^ on CD8^+^ (left panel) and CD4^+^ T cells (*right panel*). **d** Subgroups were compared for CD8^+^ (*left panel*) and CD4^+^ (*right panel*) T cell subpopulations. Solid lines in panels (**b**-**c**) represent the median value while bars in panel (**d**) represent the median values with interquartile range. Statistical comparisons were made using Fisher’s exact probability test (**a**) and Mann-Whitney test (**b**-**d**)

As shown in Fig. 3a, subjects with activation burden = 0 (no severe immune abnormalities) were more likely to have IgM in comparison to subjects with moderate (1-2, *p-*value = 0.03) or high (>3, *p-*value = 0.18) activation burden. Conversely, no differences with respect to the presence of IgG or IgA were detected among subjects with different activation burden.

We next studied the association of anti-Tat isotypes with single T cell abnormalities. The presence of IgM and/or IgG was neither associated with alteration of CD4:CD8 ratio (Fig. 3b) nor with differences in the expression of activation and maturation markers (Fig. 3c-d), except for IgG responders who showed the lowest percentage of CD45RO^+^CD27^+^CD4^+^ T cells (*p-*value = 0.027 compared to anti-Tat antibody negative subjects). Conversely, the presence of anti-Tat IgA was associated with higher percentages of HLA-DR^+^ CD38^+^ CD8^+^ T cells when compared to anti-Tat antibody negative individuals (Fig. 3c, *p-*value = 0.01).

In summary, these data show that anti-Tat of the IgM isotype were associated with a low activation burden while anti-Tat IgA were associated with high CD8^+^ T cells activation.

### Concurrence of anti-Tat IgG and IgM protects from disease progression

**Fig. 4:**
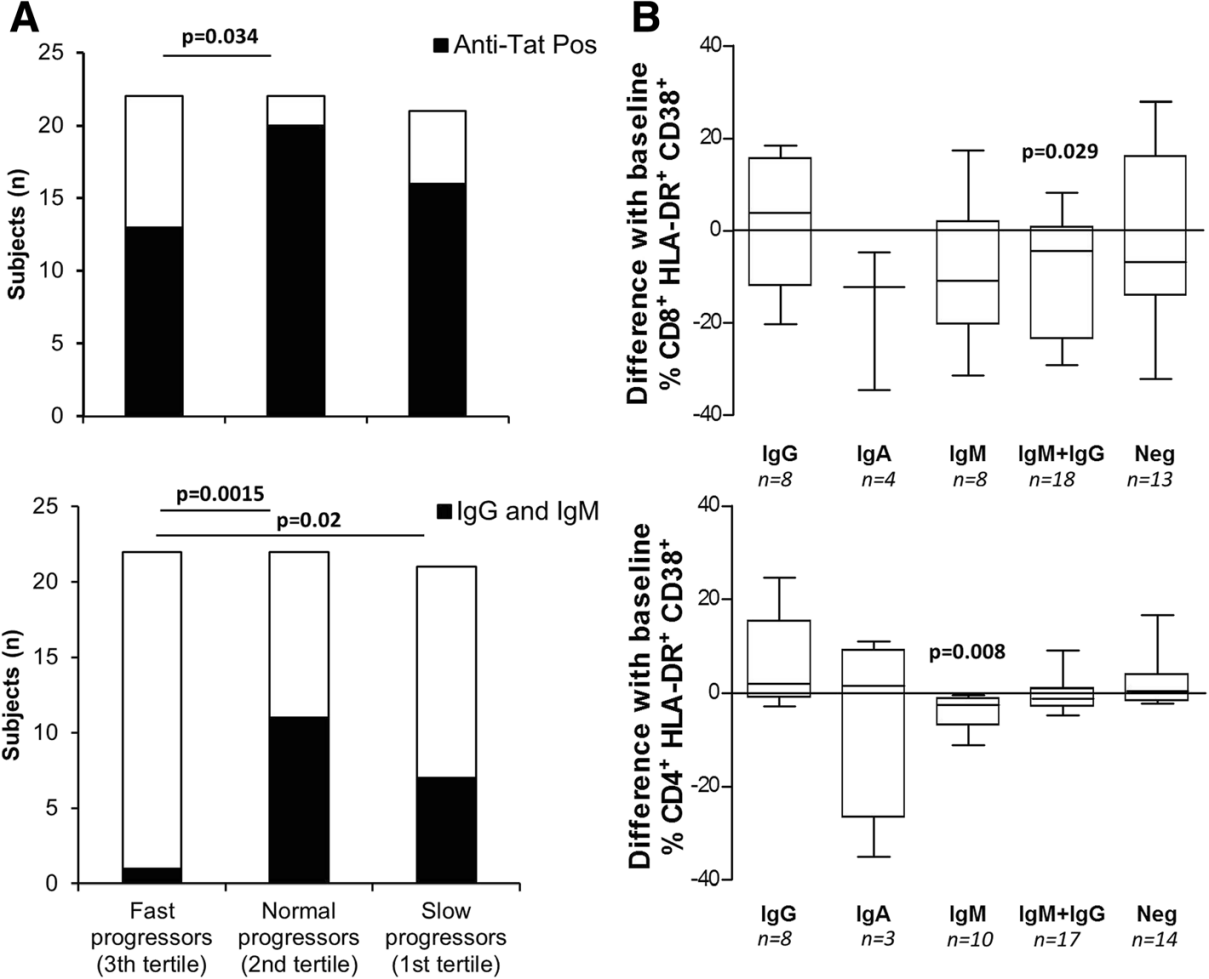
Association of different anti-Tat antibody isotypes with disease progression. **a** Subjects were stratified according to the tertiles of CD4^+^ T cell decline. Black bars represent subjects with any anti-Tat antibody isotype (top panel) or with anti-Tat IgG and IgM (bottom panel). Statistical comparisons were made using Fisher’s exact probability test. **b** Subjects were stratified according to the type and number of anti-Tat antibody isotypes. Subgroups were compared for absolute differences of CD38^+^HLA-DR^+^CD8^+^ T and CD38^+^HLA-DR^+^CD4^+^ T cell percentages. Absolute differences were obtained by subtracting baseline values from values collected at 1 year follow up visit. Data are presented as Box-and-Whisker Plots. Statistical significance of absolute difference was calculated using Wilcoxon signed rank test; *p-*values < 0.05 indicates that differences are significantly different from 0

To determine whether the presence of anti-Tat antibodies could protect against AIDS progression, CD4^+^ T cell counts were measured 1 year after the baseline anti Tat measurements. These data were available for 65 of the 96 subjects (67.7 %). For these individuals we calculated the difference in CD4^+^ T cell counts measured at baseline and after 1 year follow up, expressed as a percentage decrease (or increase) and grouped the subjects into tertiles based on this measure [40, 41]. Subjects with the lowest CD4 T cell counts (third tertile) experienced the fastest CD4^+^ T cell decline (median change: -25.2 %). Those in the middle tertile showed a moderate decrease or stable CD4^+^ T cell count (median change: -8.8 %), while those with the highest CD4 T cell counts at baseline (first tertile) generally had an increased CD4^+^ T cell count (median change: +19.6 %). As shown in Fig. 4a, subjects with the fastest CD4^+^ T cell decline (lowest tertile) were more likely to be anti-Tat antibody negative (*p-*value = 0.034 vs middle tertile). Moreover, individuals with both anti-Tat IgG and IgM were significantly more represented in the middle (*p-*value = 0.0015 vs lowest tertile) and upper (*p-*value = 0.02 vs lowest tertile) tertiles, with only one subject present in the lowest tertile. Notably, these subjects also experienced a significant decrease of the HLA-DR^+^CD38^+^CD8^+^T cell percentages during this time interval (Fig. 4b, *p-*value = 0.029).

Together, these results are consistent with the concurrence of anti-Tat IgM and IgG being protective with respect to CD4^+^ T cell decline and exacerbation of immune activation.

## Discussion

Anti-Tat antibodies have been shown to be associated with slower progression to AIDS [19, 23, 24, 42], and vaccination with Tat protected HIV-infected subjects from CD4^+^ T cells decline and immune dysfunction [26, 28, 29]. However little is known about the interplay of different anti-Tat antibody isotypes in HIV control and their association with immunological abnormalities, especially in non-clade B cohorts.

In this cohort of chronically HIV-infected subjects from South West Tanzania, anti-Tat IgM and IgG showed a similar prevalence (~50 %), while anti-Tat IgA were detected in only 15 % of subjects. Data from clade B cohorts show the frequency of anti-Tat IgG to be ~20 % [17, 21, 43], although higher frequencies have been reported [23, 44], conceivably depending on the cohort and the assay used to detect anti Tat antibodies.

Tat is a highly conserved protein, not only between different isolates of the same clade, but also across clades, and the highest levels of similarity are found in domains essential for Tat functions and in those containing immunodominant epitopes [43, 45]. Consistently with this and with reports showing that anti-Tat antibodies elicited against Tat expressed by one HIV clade may recognize Tat from different HIV clades [43, 46–48], our data demonstrate that a high proportion of individuals with detectable anti-Tat antibodies were able to recognize both clade B and C Tat. This was observed particularly for IgM. However, ELISA tests performed to measure anti-clade B Tat IgM displayed background levels that were nearly double of those observed measuring anti-clade C Tat IgM (Additional file 2C). High noise signals that interfered with the detection of anti-clade B Tat IgM have also been reported by other groups and explained as a cross-recognition of some endogenous peptides with sequences similar to clade B Tat [18, 49]. Thus, we cannot exclude that, among donors with IgM recognizing anti-clade B Tat, some may actually have antibodies directed against endogenous epitopes and cross-reacting with Tat. However, the fact that these subjects are also positive for anti-clade C Tat IgM, whose background levels are low and similar to those observed for IgA or IgG detection, would argue in favour of the response observed being a truly anti-clade B Tat IgM.

Interestingly, some subjects not recognizing clade C Tat had antibodies against clade B Tat, a subtype absent in the Mbeya region [31–33]. These subjects could have been infected either with a clade D subtype, which shares a common ancestry with clade B [50, 51], or with HIV-1 from other clades that share certain epitope-sequences closely related to the tested B sequence.

Chronically HIV-infected individuals with anti-Tat IgM had relatively high CD4^+^ T cell counts and low viral load. Anti-Tat IgM was still detectable after several years of infection and the duration of infection did not affect the association of IgM with slow disease progression. The persistence of IgM during chronic infection is intriguing and recently described for other infections [52, 53]. Moreover, anti-Tat IgM has been observed to also persist in Tat-vaccinated subjects [27], suggesting that Tat-specific IgM+ memory B cells are long lived. IgM are highly efficient in activating the complement system and in inhibiting virus entry by directly interacting with HIV co-receptors [54]. Although further studies are needed to determine the precise role of long-lived anti-Tat IgM, this isotype has been shown in different context to be highly cross-reactive, protective and to sustain IgG responses [52, 55, 56]. Moreover, the cross-recognition of self peptides by anti-Tat IgM [18, 49] may constitute a potential mechanism of protection as IgM autoantibodies have been shown to prevent excessive inflammation [57]. Consistently, we observed less pronounced T cell abnormalities in subjects with anti-Tat IgM, who prospectively showed a decrease of HLA-DR^+^CD38^+^ and CD45RO^+^CD27^+^ CD4^+^ T cells (Fig. 4b and Additional file 4 respectively), a cell subset containing central and transitional memory cells (important viral reservoirs) and whose increased percentage correlated with progression to AIDS (data not shown). Together, these data suggest that the presence of anti-Tat IgM may counteract disease progression and, in accordance with reports from European and American cohorts [17, 18, 22], this effect is independent of the HIV clade. In addition, the titer of anti-Tat IgM inversely correlated with the levels of CD8^+^ T cells with an effector memory-like phenotype (CD45RO^+^CD27^−^, Additional file 5), a subpopulation induced by Tat [8, 9].

Individuals positive for anti-Tat IgM and developing IgG responses were protected from rapid CD4^+^ T cell decline. However, anti-Tat IgG prevalence did not differ between patients stratified according to CD4^+^ T cell counts, in contrast to observations made with clade B HIV infected individuals [17, 18]. This implies that an association of anti-Tat IgG with progression to AIDS could depend on: i) the HIV clade and/or ii) the presence of multiple anti-Tat isotypes. Tat is a largely unstructured and pleiotropic protein formed by several domains that have different role with respect to HIV replication in infected CD4^+^ T lymphocytes and immunomodulatory effects on uninfected cells [2, 58]. Small differences at the levels of these domains between clade B and clade C Tat may alter its transcriptional activity and/or immunomodulatory properties [46, 59–61]. Thus, IgG-mediated neutralization of Tat in clade B and C HIV-infected individuals may have different clinical outcomes. In addition, mutations observed between the two Tat variants influence the net charge and the isoelectric point [62], inducing local structural variations [60, 61, 63] and thus potentially affecting conformational epitopes. Indeed, clade B Tat is more immunogenic in animals, compared to other Tat clades [46], and we cannot exclude that during natural infection clade C Tat induces IgG directed towards irrelevant or non-neutralizing epitopes, despite relatively high levels of binding antibodies cross-recognizing the whole protein. Further investigations including a proper mapping of conformational epitopes and neutralization or functional assays may help to clarify the potential synergy or interference between different antibody isotypes.

The role of serum HIV-specific IgA has been debated before. While some reported that serum IgA may display neutralizing activity [35], results from the recent RV144 trial demonstrated that serum anti-Env IgA may counteract the activity of protective IgG [64]. HIV-infected individuals with anti-Tat IgA displayed significantly higher pVL and activation of CD8^+^ T cells and lower CD4^+^ T cell counts. No evidence of accelerated progression in these subjects was found, although the follow up period was limited (1 year). Together with the fact that anti-Tat IgA were almost absent in patients infected for less than 3 years, this observation indicates that this isotype may not necessarily favor disease progression but rather represents a marker of late progression.

## Conclusions

This study characterizes for the first time different anti-Tat antibody isotype responses in relation to HIV disease progression in an African cohort. Although additional longitudinal studies are needed to determine the stability and persistence of the different anti-Tat antibody isotypes and their relationship with HIV disease progression, our data suggest that anti-Tat antibodies are more prevalent in this non-clade B HIV -infected cohort as compared with clade B HIV-infected cohorts.

We observed that serum anti-Tat IgA are associated with high viral load, low CD4^+^ T cell counts and high immune activation. Others have already proposed serum IgA as marker of antiretroviral therapy failure [65], and our results suggest its use to monitor late stages of disease even in untreated subjects.

Anti-Tat IgM was associated with slow disease progression, and this effect was independent of the duration of infection. Contrary to observations made with clade B HIV-infected individuals [20–22], anti-Tat IgG alone did not confer advantages in terms of better prognosis, but the concurrent presence of IgG and IgM was associated with a slower CD4^+^ T cell decline. The identification of differences in anti-Tat antibody effector functions and epitope specificity in subjects infected by different HIV clades may provide further clues for inducing/boosting effective anti-Tat responses to control HIV infection. Our data show that anti-clade C Tat immunity is associated with slow disease progression but is less protective than anti-clade B Tat immunity.

Injection of clade B Tat induced protective responses in HIV-1 clade B infected subjects [26, 27]. In addition, clade B Tat is highly cross-recognized and induces cross-reactive antibodies [43, 46–48]. Based on our and others’ findings, we consider the enhancement of anti-Tat immunity as a promising immunotherapeutic strategy for HIV-infected individuals. To achieve this goal, vaccination with cross-reactive proteins from heterologous clades should be further investigated, such as the use of clade B Tat in clade C HIV-infected cohorts.

## Abbreviations

ABTS, 2,2′-Azino-bis (3-ethylbenzothiazoline-6-sulfonic acid); AIDS, acquired immune deficiency syndrome; BSA, bovine serum albumin; cART, combination antiretroviral therapy; ELISA, enzyme-linked immunosorbent assay; HIV, human immunodeficiency virus; HRP, horseradish peroxidase; Ig, immunoglobulin; OD, optical density; pVL, plasmatic viral load; WHIS, Worm_HIV_Interaction_Study

## Additional files

Additional file 1: Alignment of used clade B and C Tat sequences. (DOCX 43 kb) Additional file 2: magnitude of anti-Tat humoral responses: (A) Anti-clade B and C Tat IgG, IgA or IgM titers were determined in anti-Tat positive sera and displayed for every donor. (B) Heatmap showing, for single donors, positivity (black) or negativity (grey) towards clade C or clade B Tat for each isotype. (C) Optical density (O.D.) from screening ELISA tests are shown for each donor after cut-off subtraction. Cut-off values were included in the following ranges: 0.128 ± 0.029 for anti-clade C Tat IgG; 0.1245 ± 0.042 for anti-clade B Tat IgG; 0.1177 ± 0.022 for anti-clade C Tat IgA; 0.1387 ± 0.031 for anti-clade C Tat IgA; 0.1963 ± 0.060 for anti-clade C Tat IgM; 0.370 ± 0.086 for anti-clade B Tat IgM. (JPG 154 kb) Additional file 3: Frequencies of CD4^+^ and CD8^+^ T cells expressing activation and maturation markers in the peripheral blood: Shown are representative plots demonstrating the gating strategy for the expression of activation (CD38 and HLA-DR; left panel) and maturation (CD27 and CD45RO; right panel) markers on CD4^+^ (upper panels) and CD8^+^ (lower panels) T cells. (JPG 316 kb) Additional file 4: Prospective analysis of the association of different anti-Tat antibody isotypes with T cell subpopulations. Subjects were stratified according the type and number of anti-Tat antibody isotypes. Bars show the proportion of the different subsets in the CD8^+^ and CD4^+^ T cell compartments, based on the median value of each subset measured at baseline and after 1 year. For each subset, differences from baseline were calculated using Wilcoxon signed rank test. + and - indicate respectively significant increases and decreases with p-values < 0.05. (JPG 231 kb) Additional file 5: Correlation between anti-clade B and C Tat IgM titers and % of Effector Memory CD8^+^ T cells: IgM titers from anti-clade B and C Tat IgM responders (upper and lower panel, respectively) were plotted against percentage of effector CD45RO^+^CD27^−^ CD8^+^ T cells. Correlation was assessed by Spearman r. (JPG 182 kb)
