# Supplementary material for: Association between different anti-Tat antibody isotypes and HIV disease progression: data from an African cohort
Source: BMC Infect Dis. 2016 Jul 22;16:344. doi: 10.1186/s12879-016-1647-3 (PMC4957276; doi:10.1186/s12879-016-1647-3)
Supplement: Additional file 1: — Alignment of used clade B and C Tat sequences. (DOCX 43 kb) [file 12879_2016_1647_MOESM1_ESM.docx]

**Additional File 1**

10 20 30 40 50 60 70 80 90 100

....|....|....|....|....|....|....|....|....|....|....|....|....|....|....|....|....|....|....|....|..

AAA44199.1 (B) **MEPVDPRLEPWKHPGSQPKTACTNCYCKKCCFHCQVCFITKALGISYGRKKRRQRRRPPQGSQTHQVSLSKQPTSQSRGDPTGPKE---------------**

AAL06113.1 (C) **......N....N..........NT......SY..L...Q..G..............SA.PS.ED..NPI....LPRTQ.....SE.SKKKVESKTETDPFD**
