# Supplementary figures and images for: Association between different anti-Tat antibody isotypes and HIV disease progression: data from an African cohort
Source: BMC Infect Dis. 2016 Jul 22;16:344. doi: 10.1186/s12879-016-1647-3 (PMC4957276; doi:10.1186/s12879-016-1647-3)

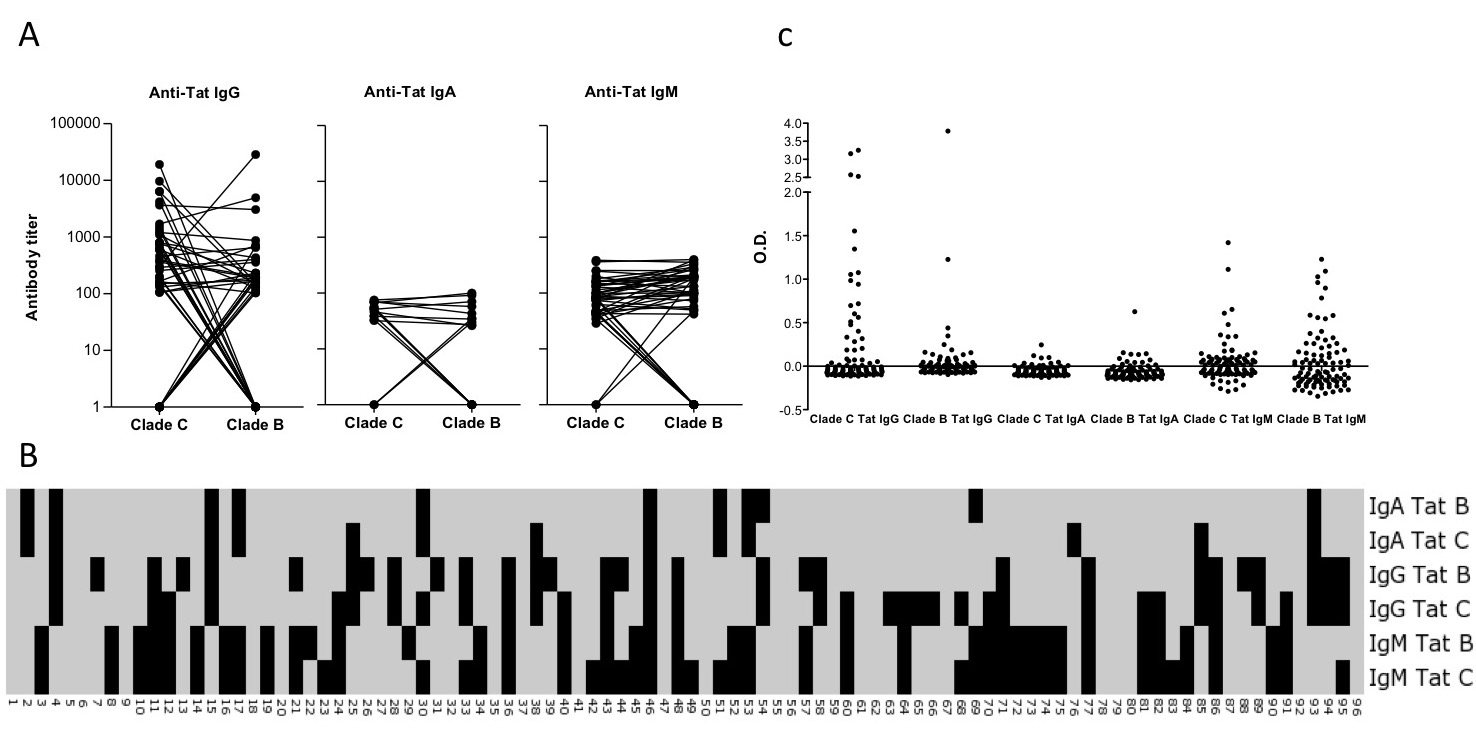

Supplement: Additional file 2: — magnitude of anti-Tat humoral responses: (A) Anti-clade B and C Tat IgG, IgA or IgM titers were determined in anti-Tat positive sera and displayed for every donor. (B) Heatmap showing, for single donors, positivity (black) or negativity (grey) towards clade C or clade B Tat for each isotype. (C) Optical density (O.D.) from screening ELISA tests are shown for each donor after cut-off subtraction. Cut-off values were included in the following ranges: 0.128 ± 0.029 for anti-clade C Tat IgG; 0.1245 ± 0.042 for anti-clade B Tat IgG; 0.1177 ± 0.022 for anti-clade C Tat IgA; 0.1387 ± 0.031 for anti-clade C Tat IgA; 0.1963 ± 0.060 for anti-clade C Tat IgM; 0.370 ± 0.086 for anti-clade B Tat IgM. (JPG 154 kb) [file 12879_2016_1647_MOESM2_ESM.jpg]

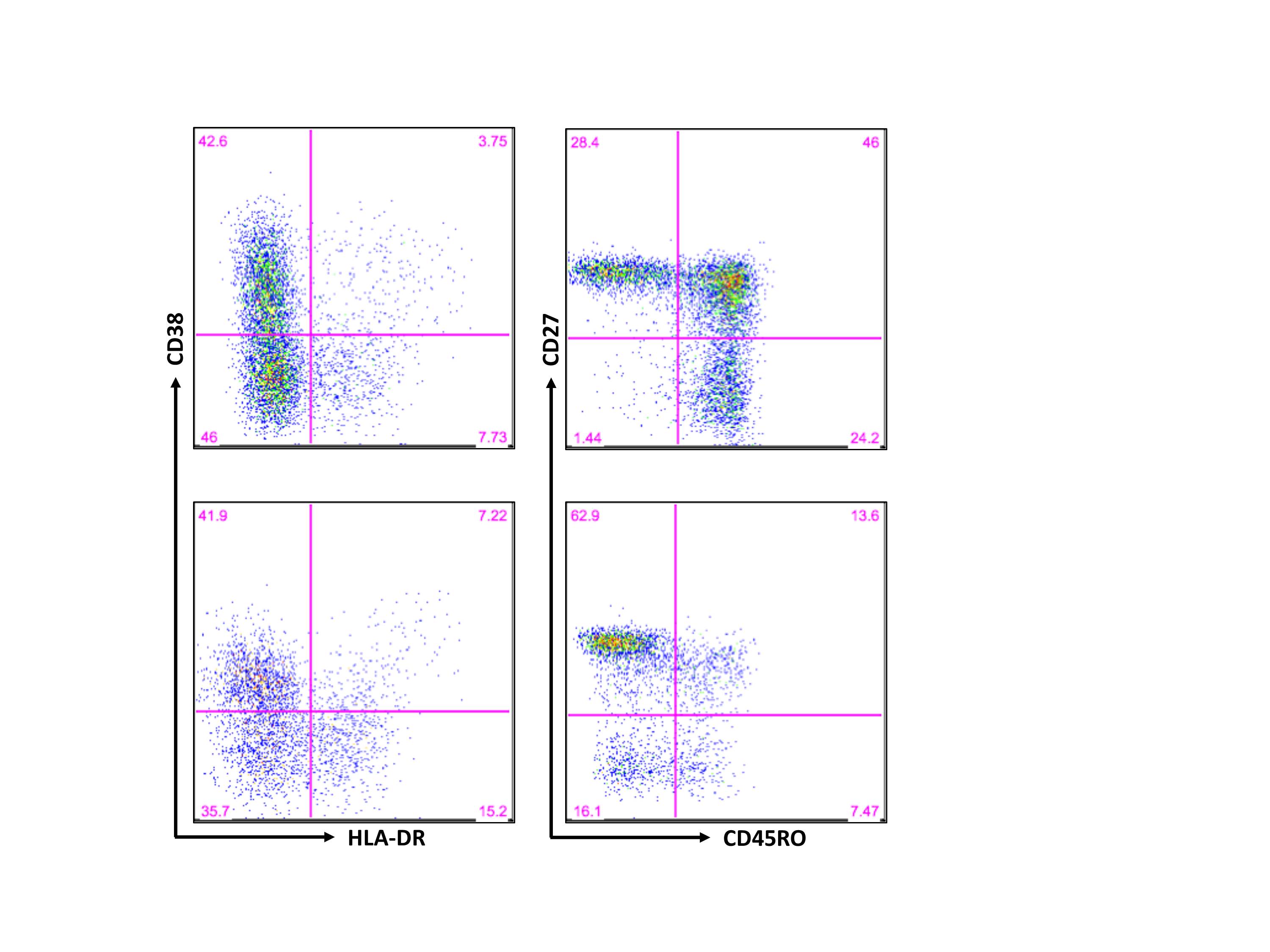

Supplement: Additional file 3: — Frequencies of CD4+ and CD8+ T cells expressing activation and maturation markers in the peripheral blood: Shown are representative plots demonstrating the gating strategy for the expression of activation (CD38 and HLA-DR; left panel) and maturation (CD27 and CD45RO; right panel) markers on CD4+ (upper panels) and CD8+ (lower panels) T cells. (JPG 316 kb) [file 12879_2016_1647_MOESM3_ESM.jpg]

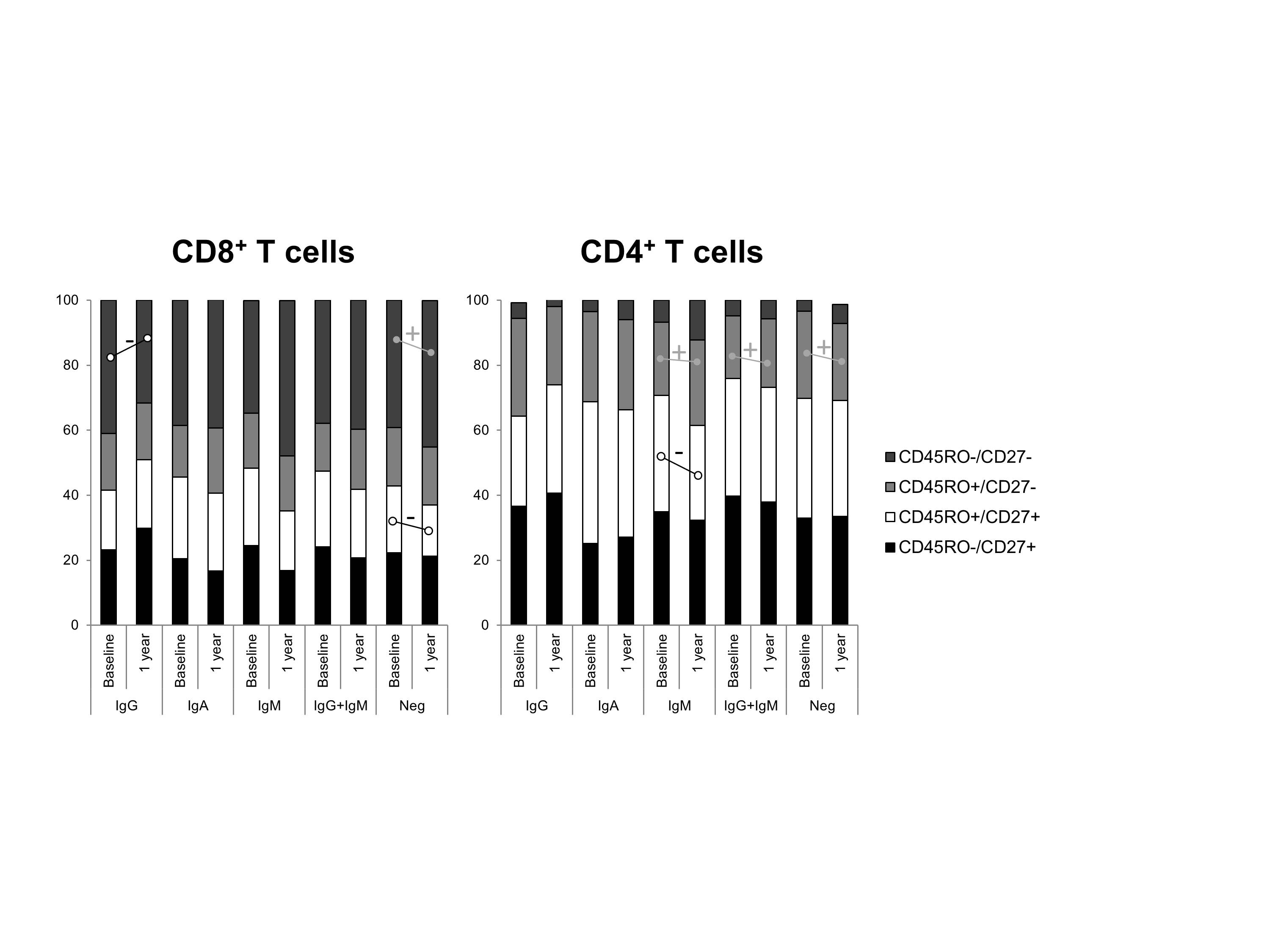

Supplement: Additional file 4: — Prospective analysis of the association of different anti-Tat antibody isotypes with T cell subpopulations. Subjects were stratified according the type and number of anti-Tat antibody isotypes. Bars show the proportion of the different subsets in the CD8+ and CD4+ T cell compartments, based on the median value of each subset measured at baseline and after 1 year. For each subset, differences from baseline were calculated using Wilcoxon signed rank test. + and - indicate respectively significant increases and decreases with p-values < 0.05. (JPG 231 kb) [file 12879_2016_1647_MOESM4_ESM.jpg]

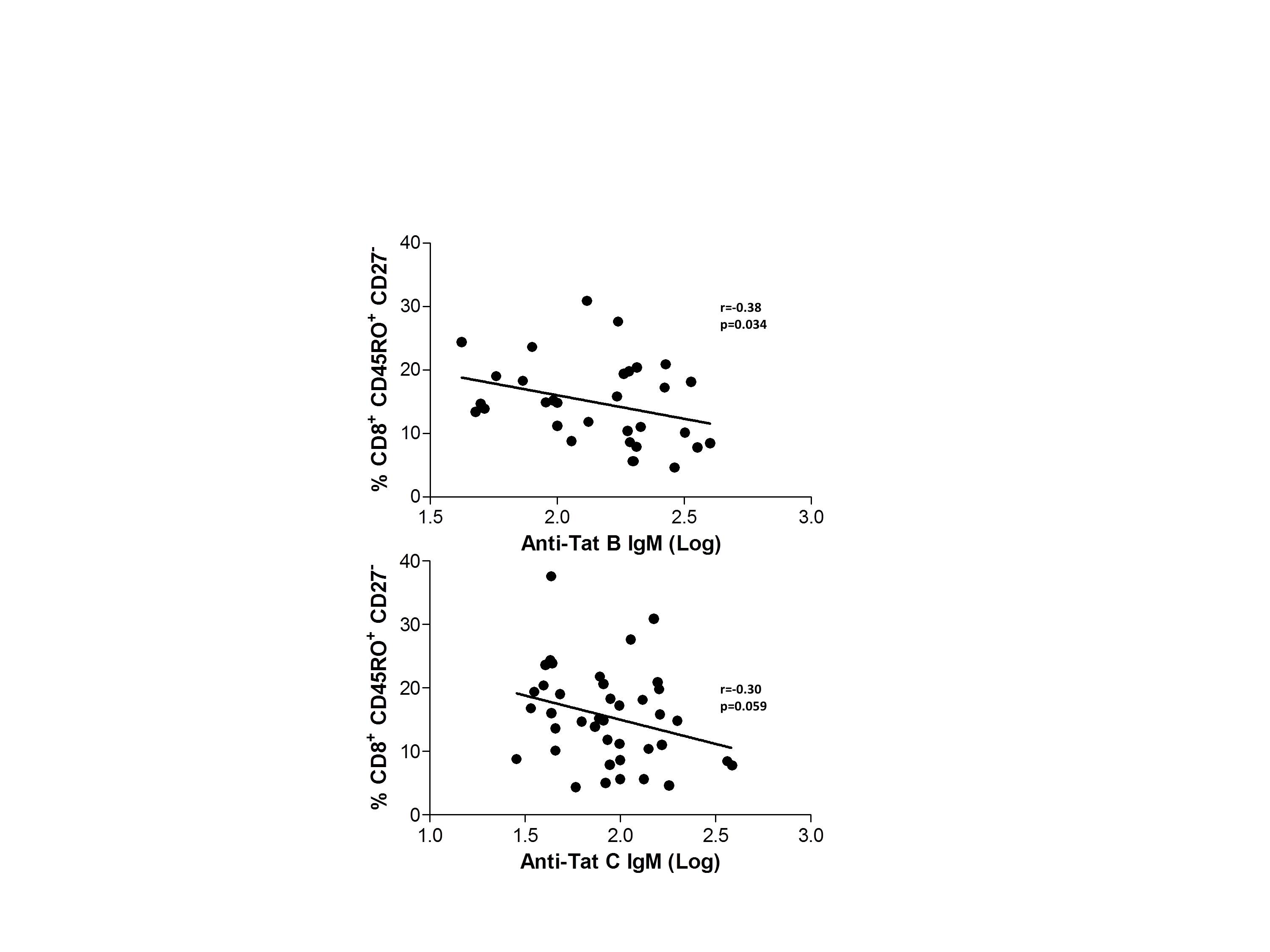

Supplement: Additional file 5: — Correlation between anti-clade B and C Tat IgM titers and % of Effector Memory CD8+ T cells: IgM titers from anti-clade B and C Tat IgM responders (upper and lower panel, respectively) were plotted against percentage of effector CD45RO+CD27− CD8+ T cells. Correlation was assessed by Spearman r. (JPG 182 kb) [file 12879_2016_1647_MOESM5_ESM.jpg]
